# The R148.3 Gene Modulates *Caenorhabditis elegans* Lifespan and Fat Metabolism

**DOI:** 10.1534/g3.117.041681

**Published:** 2017-06-15

**Authors:** Catherine Roy-Bellavance, Jennifer M. Grants, Stéphanie Miard, Kayoung Lee, Évelyne Rondeau, Chantal Guillemette, Martin J. Simard, Stefan Taubert, Frédéric Picard

**Affiliations:** * Quebec Heart and Lung Research Institute, G1V 4G5, Canada; † Faculty of Pharmacy, Laval University, Québec, G1V 0A6, Canada; ‡ Centre for Molecular Medicine and Therapeutics, University of British Columbia, Vancouver, V5Z 4H4, Canada; § British Columbia Children’s Hospital, Vancouver, V6H 3N1, Canada; ** Department of Medical Genetics, University of British Columbia, Vancouver, V6H 3N1, Canada; †† Centre Hospitalier de l’Université Laval (CHU) de Québec Research Center, G1V 4G2, Canada; ‡‡ Faculty of Medicine, Laval University, Québec, G1V 0A6, Canada

**Keywords:** *daf-2*, R148.3, insulin signaling, lifespan, fat metabolism

## Abstract

Despite many advances, the molecular links between energy metabolism and longevity are not well understood. Here, we have used the nematode model *Caenorhabditis elegans* to study the role of the yet-uncharacterized gene R148.3 in fat accumulation and lifespan. In wild-type worms, a R148.3*p*::GFP reporter showed enhanced expression throughout life in the pharynx, in neurons, and in muscles. Functionally, a protein fusing a predicted 22 amino acid N-terminal signal sequence (SS) of R148.3 to mCherry displayed robust accumulation in coelomyocytes, indicating that R148.3 is a secreted protein. Systematic depletion of R148.3 by RNA interference (RNAi) at L1 but not at young-adult stage enhanced triglyceride accumulation, which was associated with increased food uptake and lower expression of genes involved in lipid oxidation. However, RNAi of R148.3 at both L1 and young-adult stages robustly diminished mean and maximal lifespan of wild-type worms, and also abolished the long-lived phenotypes of *eat-2* and *daf-2/InsR* mutants. Based on these data, we propose that R148.3 is an SS that modulates fat mass and longevity in an independent manner.

It is well established that age-associated excessive adipose tissue accumulation is detrimental to health in humans, notably because of its negative impact on comorbidities such as type 2 diabetes, hypertension, and dyslipidemia (Carter *et al.* 2013). Discovering how increased adiposity impairs organismal health is thus of great biomedical relevance. Model organisms such as *Caenorhabditis elegans* are well suited to experimentally dissect such relationships. Nevertheless, the reciprocal interactions between aging and fat accumulation in worms remain incompletely understood and are complicated by ectopic lipid deposition in aging animals (Ackerman and Gems 2012; Hou and Taubert 2012; Palikaras *et al.* 2017).

Large screens of long-lived *C. elegans* mutants have unexpectedly showed widely varying levels of fat storage. On the one hand, long-lived *daf-2* and *glp-1 C. elegans* mutants show increased fat accumulation compared to wild-type worms (O’Rourke *et al.* 2009). Moreover, inhibition of autophagy, which results in a shortened lifespan, also leads to a reduction in stored lipids (Lapierre *et al.* 2013, 2011), in part through modifications in lysosomal lipid degradation (O’Rourke and Ruvkun 2013). On the other hand, akin to preclinical observations (Mattison *et al.* 2017), *eat-2* mutants, also long-lived, have lower fat accumulation due to calorie restriction (Lakowski and Hekimi 1998). Similarly, activation of *skn-1* protects against high carbohydrate diet-induced fat accretion through the transcriptional cofactor *mdt-15*, which increases longevity (Pang *et al.* 2014; Taubert *et al.* 2006). These findings suggest that the regulation of longevity and lipid metabolism is complex and involves multiple genes and signaling pathways.

We serendipitously found that loss of the R148.3 gene in *C. elegans* resulted in a short lifespan. In this context, the aim of this study was to test whether R148.3 integrates the control of longevity with lipid storage. We observed that loss of the R148.3 gene increased lipid accumulation and robustly shortened lifespan in an apparently dissociated manner, possibly by acting as a secreted signaling molecule.

## Materials and Methods

### Strains

*C. elegans* strains were maintained and studied as described (Brenner 1974) on solid nematode growth media (NGM) at 20°, except when stated otherwise. Wild type was the N2 Bristol strain, and the following mutants were used: *daf-2*(*e1370*) *III*, *daf-16*(*mu86*) *I*, *eat-2*(*ad465*) *II*, and *sbp-1*(*ep79*) *III*. Strains AGP24f glmEx5 (*Pnhr-49*::*nhr-49*::*GFP* + *Pmyo-2*::*mCherry*) (Ratnappan *et al.* 2014) and TJ356 [*zIs356* (*daf-16p*::*daf-16a/b*::*GFP* + *rol-6*)] (Henderson and Johnson 2001) were described previously. NHR-49::GFP transgenics were selected among those expressing a *Pmyo-2*::*mCherry* co-injection marker (Ratnappan *et al.* 2014). For all experiments, synchronization was obtained by bleaching (alkaline hypochlorate method). Strains and reagents are available upon request.

### Quantitative real-time PCR

Worms were collected and washed thoroughly with 1× M9 buffer. Depending on the developmental stage, 100–400 worms were used for each assay. Total RNA was extracted according to the standard protocol provided with RNAspin Mini Isolation Kit (GE Healthcare, Baie d’Urfe, QC, Canada), except for the first step, during which samples were vortexed in lysis buffer with β-mercaptoethanol and kept at room temperature for 10 min, followed by a series of three heat shocks at 42° and an additional 30-sec vortex step. Worm lysis was assessed by microscopy. RNA was quantified with the NanoVue system (GE healthcare). 1 µg of RNA was used for reverse transcription using the qScript cDNA SuperMix (Quanta) following the manufacturer’s instructions. Quantitative real-time PCR (qPCR) was performed using SYBR Green PCR Master Mix without MgCl_2_ (Invitrogen). Data were analyzed using the standard curve method and normalized with expression values of the housekeeping gene *act-1*. Primers used in this study are presented in Supplemental Material, Table S1 in File S2.

### R148.3p::GFP construction

The proximal 1.5-kb portion of the R148.3 promoter sequence was obtained by PCR using N2 genomic DNA as template and cloned into the pFX-EGFPT vector. Primer sequences used were: 5′-AATTTCGCGATTTTTGAACA-3′ and 5′-CATTATATACAACGCGGAGT-3′. The transgenic strain was generated by micro-injecting a mix containing this expression plasmid along with pRF4 [*rol-6*(*su1006*)] as co-injection marker and UV integrated as described (Mello *et al.* 1991; Mello and Fire 1995). F2+ progeny displaying the Roller phenotype were selected.

### R148.3ss (myo-3p::SS_R148.3_::mCherry)

The putative R148.3 signal sequence (R148.3ss) was inserted into the pCFJ104 [*myo-3P*::*mCherry*] vector using the NEB Q5 Site-Directed Mutagenesis Kit (E0554), with the primers fwd_myo-3P-R148.3ss (5′-TTTATCTCTGAAAAGCAGTGTTCC-3′) and rev_myo-3P-R148.3ss (5′-AGAGCATGTAGGGATGTTGAAG-3′). The resulting plasmid, or an unmutagenized pCFJ104 [*myo-3P*::*mCherry*] control, were micro-injected into the gonads of wild-type *C. elegans* (5 ng/μl final concentration) with 100 ng/μl of pRF4 co-injection marker and 95 ng/μl of pPD95.77 empty vector. Two independent transgenic lines were isolated by selecting F2 progeny displaying the Roller phenotype.

### R148.3 RNAi

PCR amplification of a sequence targeting R148.3 was performed using primer sequences 3′-attgctaatacgactcactatagggTCTAGAaagagcccctagagccagtc-5′ and 5′-attgctaatacgactcactatagggGGTACCctcgggctcttcaacttttg-3′, and the resulting PCR product was cloned into the L4440 feeding RNAi vector. L4440-R148.3 RNAi and empty L4440 vectors were transformed into HT115 bacteria, and the resulting strains were used as food source for *C. elegans* feeding RNAi experiments. RNAi bacteria were cultured for 16 hr in LB containing 100 µg/ml ampicillin and seeded on RNAi NGM agar plates containing 1 mM isopropylthiogalactoside (IPTG) and 100 µg/ml carbenicillin. Plates were incubated overnight at room temperature to induce dsRNA expression. For specific induction of RNAi at the L1 stage, eggs were placed on bacteria expressing the different RNAi clones. For induction of RNAi at L4/young-adult stage, synchronized L4 larvae were placed on bacteria expressing the different RNAi clones. To study effects of R148.3 RNAi in larvae (gene expression and lipid staining), F2+ generations of parent worms continuously fed with the different clones were used.

### Pharyngeal pumping assay

Synchronized L1 larvae were transferred onto RNAi plates and cultured at 20° for 4 d. Then, five worms were selected from each group and pharyngeal pumping was counted 10 times for a period of 30 sec each for every worm.

### Life-span assay

Synchronized L1 or L4 worms (as indicated in the figures) were grown on bacteria expressing the different RNAi clones. L1 plating was considered day 1 of the experiment. Worms were transferred to a new plate every other day during the reproductive period, and every week during the postreproductive period. Animals were kept at 20° except for experiments on *daf-2* mutants, which were kept at 25° after reaching adulthood (they were kept at 16° until adulthood to prevent entry into dauer stage). Animals were considered dead when they failed to respond to prodding. Animals that crawled off the plates, died of drying on the edges of the plate, bagging, or vulva bursting were excluded from all experiments. Tables S3–S9 in File S2 detail the life-span data for each experiment.

### Fixed Oil Red O staining

100–200 synchronized worms were washed two times with M9 and then resuspended in 120 μl of M9 buffer. 120 μl of 2× MRWB buffer (160 mM KCl, 40 mM NaCl, 20 mM EGTA, 1 mM spermidine, 0.4 mM spermine, 30 mM Na•PIPES, 0.2% β-mercaptoethanol) with 2% PFA were added. Worms were then incubated for 1 hr at room temperature, washed twice with 1 ml M9, and then resuspended and incubated in 60% isopropanol for 15 min at room temperature. After washing with M9 buffer, an Oil Red O (ORO) staining solution (0.2% w/v in isopropanol) was added and the worms were incubated overnight at room temperature with agitation. The nematodes were then washed three times and observed with an Olympus MVX10 microscope. Pixel measurement from ORO stained worms was performed with ImageJ software.

### Live Nile Red staining

250 μl of Nile Red (1 μg/ml) was added on top of NGM plates already seeded with RNAi bacteria. Synchronized L1 worms were placed on these plates and day-1 adults were then washed, paralyzed, and observed under an Olympus MVX10 microscope.

### Triglyceride measurements

Triglyceride extraction was done as previously described (Barros *et al.* 2012). Synchronized worms were transferred into a 9-ml glass tube and subjected to repetitive washing with M9. Final volume was reduced to 1 ml after a wash with double-distilled water (ddH_2_O). A 0.1-ml aliquot of vortexed worm suspension was set aside for protein quantification. To the remainder, chloroform, methanol, and water were added (1:2:0.8 chloroform/methanol/ddH_2_O ratio). This mix was vortexed for 20 min at room temperature. Then, chloroform and 0.2 N HCl were added in a 1:1 ratio, followed by a second vortex session of 20 min. After phase separation (centrifugation at 1000 × *g* for 5 min), the bottom phase was removed using a Pasteur pipette and carefully transferred into a “cleaning” phase tube containing 1.2 ml ddH_2_O, 1.5 ml, 0.2 N HCl, 3 ml chloroform, and 3 ml methanol. The tube was vortexed and centrifuged at 1000 × *g* for 5 min. The top phase was removed with a Pasteur pipette and the lower phase was then evaporated with N_2_. The dried sample was resuspended in fresh isopropanol and triglycerides were quantified by colorimetry (WAKO, Richmond, VA). Data were corrected for total protein concentration, as determined using a Bio-Rad assay according to the manufacturer’s instructions.

### Lipid metabolite measurements

Synchronized L4 worms were washed three times in 10 ml cold M9 buffer. 0.1 ml of worm suspension was transferred into a tube, pelleted by centrifugation, and kept for further genomic DNA quantification. For the assay, worms were allowed to settle and the supernatant was removed. The worm pellet was resuspended in 15% (v/v) 10 mM phosphate buffer completed with 85% cold anhydrous ethanol. Samples were then subjected to 15 cycles of 15 sec 50% amplitude sonication following by freeze (liquid N_2_)/thaw (95°). Samples were then centrifuged at 16,000 × *g* for 20 min at 4°. Supernatants were transferred into cryogenic vials and kept in liquid N_2_ until time of assay.

The targeted metabolomics approach was based on electrospray ionization-tandem mass spectrometry (ESI-MS/MS) measurements using Absolute*IDQ* p150 Kits (Biocrates Life Sciences AG, Innsbruck, Austria), which allows the quantification of up to 188 endogenous metabolites from five different compound classes including acylcarnitines, amino acids, hexoses, phospho- and sphingolipids, and biogenic amines. The assay procedures and the metabolite nomenclature have been described in detail (Kastenmuller *et al.* 2011; Gieger *et al.* 2008). MS analyses were performed on a API4000 LC/MS/MS System (AB Sciex, Concord, ON, Canada) equipped with a UFLC Prominence (Shimadzu Scientific Instrument, Columbia, MD) and controlled by the software Analyst version 1.6.2. Isotope-labeled internal standards and other internal standards were integrated into a kit plate filter for metabolite quantification. The Biocrates MetIDQ software was used to control the assay workflow, from sample registration to automated calculation of metabolite concentrations and the export of data into Excel for subsequent analysis and statistics. Concentrations of all analyzed metabolites are reported in micromolar. Data were corrected for total genomic DNA concentrations, as determined using the BioDrop reader (MBI, Montreal, QC, Canada). Table S2 in File S2 details all individual metabolomics data.

### Oxygen consumption measurements

Oxygen consumption was measured in a Seahorse XFe 24 Extracellular Flux Analyzer according to the manufacturer’s instructions. Worms were rinsed twice in M9 buffer to remove bacteria and then kept on unseeded sterile agar plates. 75 worms were manually transferred into each well, containing 450 µl M9 buffer, of an XFe 24-well assay plate. Basal oxygen consumption was then determined according to the following protocol: three cycles of 1-min mix, 3-min wait period, and 3 min of measurements. For every well, oxygen consumption data were corrected by the number of worms.

### Statistical analysis

Data presented herein are from at least three independent experiments performed in triplicate of at least ∼100 worms. All data are represented by mean ± SEM. Mean survival was used to calculate effect on lifespan. *P* values for the survival curves were assessed with Kaplan–Meier survival analysis, log-rank test against controls animals using the OASIS application (Yang *et al.* 2011). *P* values for the phenotypic and metabolic traits as well as gene expression were calculated by one-way ANOVA. A *P* value < 0.05 was considered statistically significant.

### Data availability

The authors state that all data necessary for confirming the conclusions presented in the article are represented fully within the article.

## Results

### R148.3 is expressed widely and contains a functional SS for secretion

**Figure 1:**
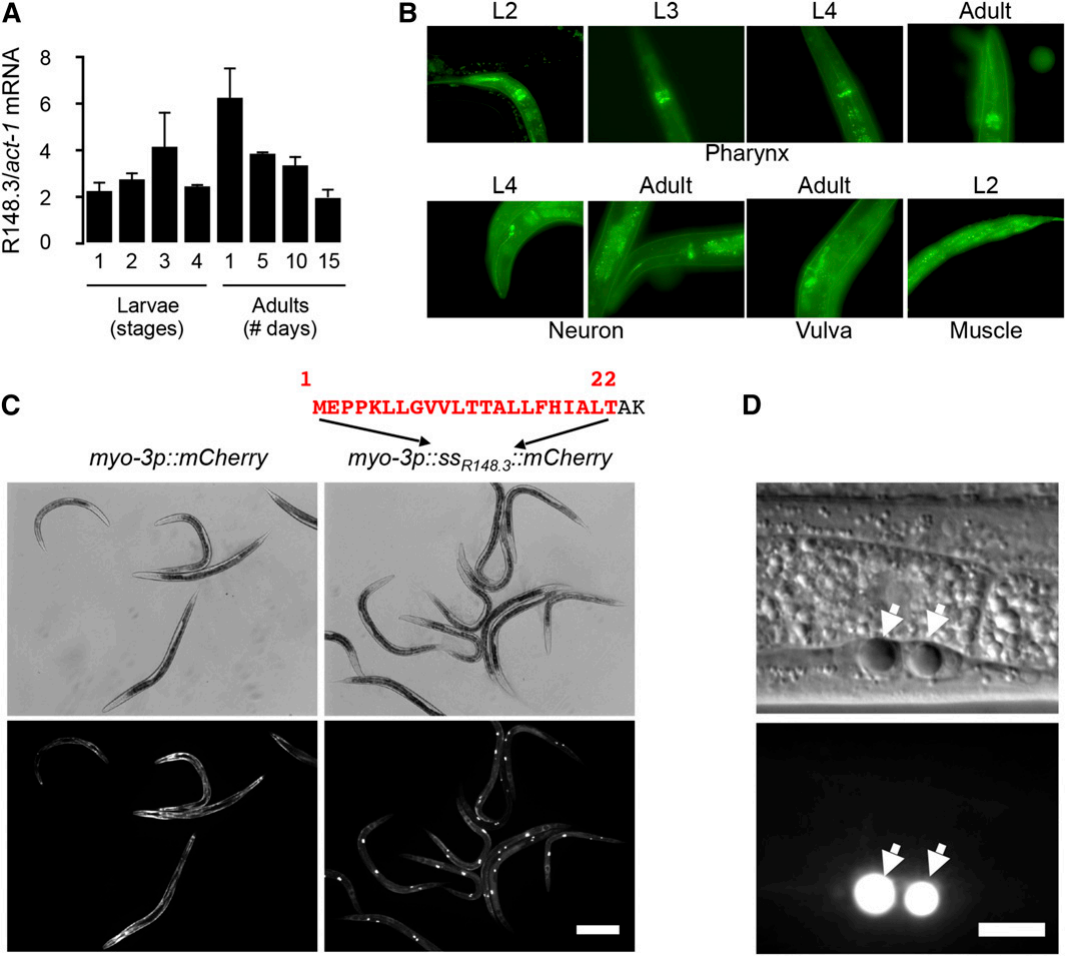
Expression of R148.3 in *C. elegans*. (A) Expression levels of R148.3 were measured by qPCR in N2 worms at different stages of their life; bars represent average ± SEM (*n* = 3). (B) Expression of a R148*p*::GFP reporter in the pharynx, neurons, vulva, and body wall muscles. Top panels: head is oriented up; lower panels: head is oriented down. (C) Representative images of mCherry fluorescence in muscle and coelomocytes in *myo-3p*::*mCherry- vs. myo-3P*::*ssR148.3*::*mCherry*-expressing worms. Exposure: 20 msec. Bar, 200 µm. (D) Representative image of *myo-3P*::*ssR148.3*::*mCherry* expression in coelomocytes. → indicates coelomocyte nuclei. Exposure: 1 msec. Bar, 10 µm. Similar findings were obtained with a second independent transgenic line.

To gain insight into the regulation and function of R148.3, we first studied its expression. Analysis of whole-body R148.3 mRNA expression by qPCR showed consistent expression throughout development and adulthood, with a moderate and statistically not significant peak of expression on the first day of adulthood (Figure 1A). A R148.3*p*::GFP transcriptional reporter consisting of 1.5 kb of the R148.3 promoter revealed tissue expression in the pharynx, in neurons, and in body wall muscles (Figure 1B). Expression in these tissues was observed throughout the lifespan. Thus, R148.3 appears to be widely and continuously expressed.

Because R148.3 has no known function, we used bioinformatic tools to identify functional domains and motifs. Interestingly, a putative SS with a high SignalP-HMM probability of 0.985 (www.cbs.dtu.dk/services/SignalP) was found in the N-terminus of R148.3, corresponding to the first 22 amino acids (Figure 1C). To test whether this putative SS can confer secretion capacity, these 22 amino acids were cloned upstream of the mCherry protein, and this fusion protein was then expressed transgenically from the muscle-specific *myo-3* promoter. As expected, a control *myo-3p*::*mCherry* protein lacking an SS was present in muscles (Figure 1C). In contrast, the *myo-3p*::*SS*_R148.3_::*mCherry* protein was observed exclusively in coelomyocytes (Figure 1, C and D), confirming that the first 22 amino acids of R148.3 represent a functional SS and suggesting that R148.3 may be a secreted protein *in vivo*.

### Loss of R148.3 increases fat accumulation

Two independent R148.3 deletion alleles (*ok3525* and *tm4390*) are both annotated as lethal and sterile, indicating that the gene is likely essential, although this possibility needs to be confirmed using outcrossed strains. For these reasons, to study the function of R148.3 *in vivo*, we depleted it systematically by feeding RNAi. Loss of R148.3 reduced egg laying and triggered a strong Bag-of-worms (Bag) phenotype, which could be abrogated by FUDR treatment, as expected (Figure S1 in File S1).

**Figure 2:**
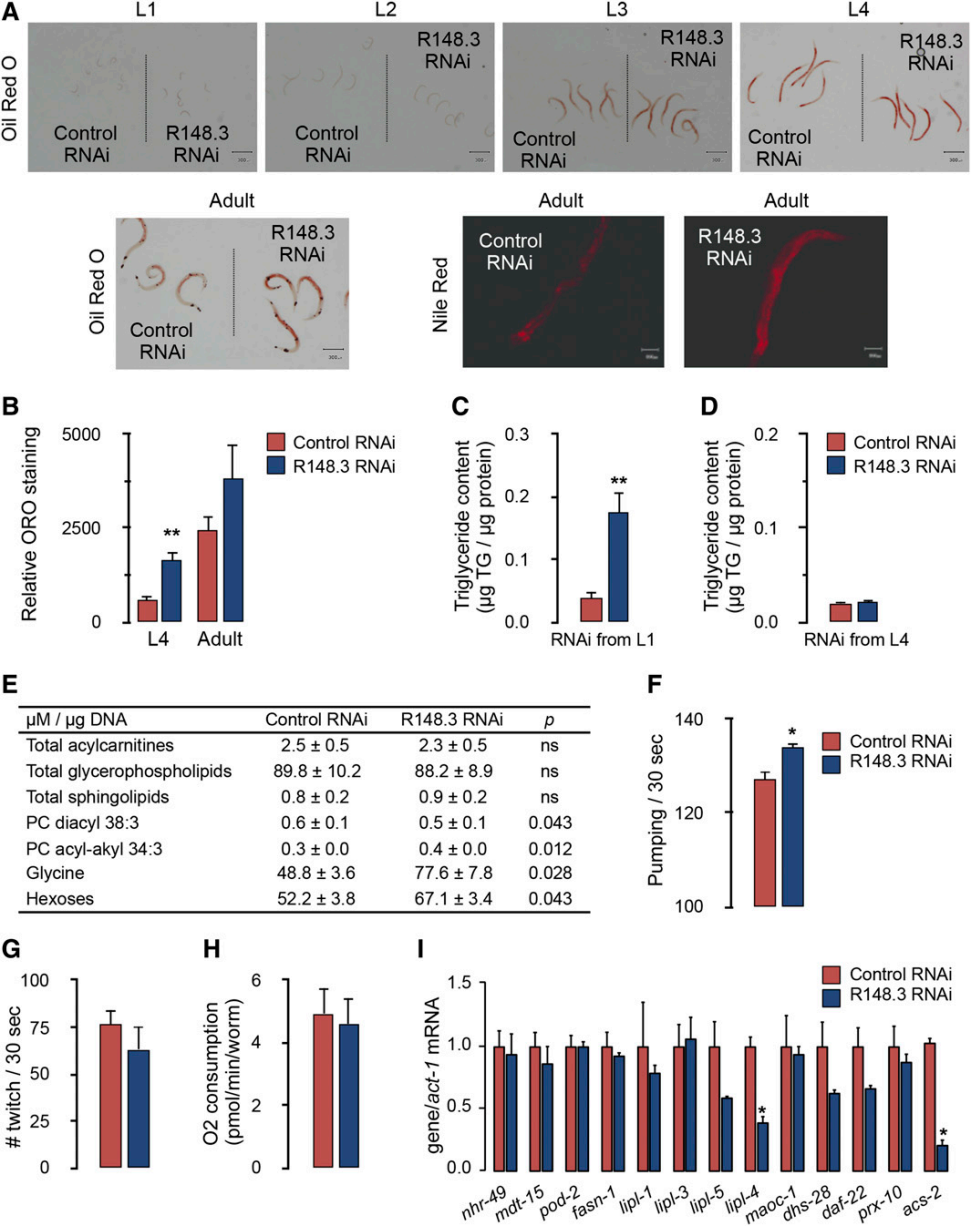
Loss of R148.3 increases triglyceride accumulation. (A) Oil red O and Nile Red staining of N2 worms fed either empty L4440 vector (control) or R148.3 RNAi starting at stage L1. Animals were assessed at all developmental stages and in adulthood. (B and C) Quantitative analysis of Oil Red O staining (B), and whole-body triglyceride content (C) of worms described in (A) and studied at L4 and adult stages. (D) Whole-body triglyceride content of worms fed either empty L4440 vector (control) or R148.3 RNAi starting at stage L4/young adult and studied 8 d later. (E) Metabolomic analysis of lipid species of worms described in (C). (F–I) Pharyngeal pumping rates (F), twitching rates (G), oxygen consumption (H), and mRNA expression levels of lipid metabolism genes (I) of L4 worms described in (C). For all panels, * *P* < 0.05, ** *P* < 0.01. Assays were performed on at least four independent plates per group, each containing 100–200 worms.

When started at the L1 stage, R148.3 depletion resulted in enhanced lipid storage in L4 and adult worms, as evidenced by qualitative Oil Red O and Nile Red staining (Figure 2A), quantitative Oil Red O staining (Figure 2B), and higher whole-body triglyceride content (Figure 2C). However, when RNAi was initiated at the L4/young-adult stage, knockdown of R148.3 did not induce triglyceride accumulation (Figure 2D). Taken together, these findings demonstrate that only reduction of R148.3 function during early larvae development, but not later in life, results in enhanced lipid accumulation.

To identify the lipid metabolites impacted by the loss of R148.3 early in life, a targeted metabolomics approach was used on L4 worms in which RNAi was initiated at stage L1. This design prevented bias due to the synthesis of egg lipids. Total levels of acylcarnitines, glycerophospholipids, and sphingolipids were similar between *control*(*RNAi*) and *R148.3*(*RNAi*) animals (Figure 2E, detailed analyses presented in Table S2 in File S2). However, R148.3 RNAi altered the levels of phosphatidylcholine (PC) diacyl 38:3 and acyl-alkyl 34:3, and robustly increased the total levels of glycine and hexoses (Figure 2E), which supports overall increased energy accumulation in worms lacking R148.3 early in life.

Energy accumulation in the form of lipids in worms in which R148.3 RNAi was initiated at stage L1 could have resulted from an imbalance between energy intake and expenditure, enhanced lipid synthesis, and/or perturbed lipid oxidation. We found that *R148.3*(*RNAi*) worms had significantly higher rates of pharyngeal pumping than wild-type worms, supporting higher food intake (Figure 2F). However, R148.3 RNAi had no impact on physical activity, as estimated by twitching (Figure 2G), nor on overall oxygen consumption (Figure 2H). Interestingly, although R148.3 RNAi did not change the relative mRNA expression of many genes involved in lipid metabolism, it strongly decreased the mRNA levels of *lipl-4* and *acs-2* (Figure 2I), two genes involved in fat hydrolysis and fatty acid oxidation, respectively. Because *acs-2* is a target of the nuclear hormone receptor NHR-49 (Van Gilst *et al.* 2005), which promotes fatty acid degradation, we examined NHR-49 expression; however, the levels and localization of an NHR-49::GFP reporter protein were not altered in *R148.3*(*RNAi*) worms (Figure 2I and Figure S2A in File S1).

### Loss of R148.3 shortens lifespan

**Figure 3:**
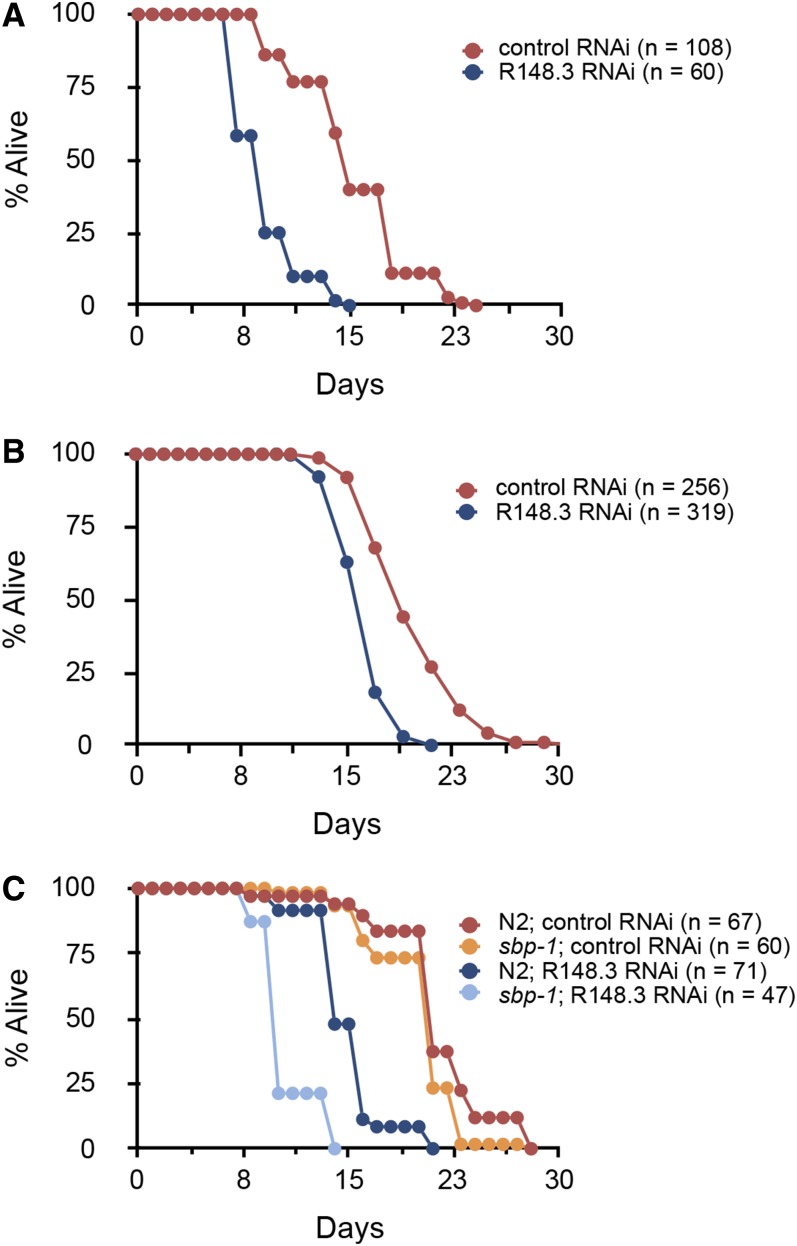
Loss of R148.3 shortens lifespan. Representative population survival curve of (A and B) N2 worms, or (C) *sbp-1* mutants fed either empty L4440 vector (control RNAi) or R148.3 RNAi. RNAi was initiated at either (A and C) stage L1 or at (B) stage L4/young adult. For all panels, *P* < 0.0001 for effects of R148.3 RNAi in all cases. See Tables S3–S5 in File S2 for additional data on replicate experiments.

Noticeably, compared to controls, worms with R148.3 RNAi initiated at the L1 stage exhibited a large (∼40%) decrease in both their mean and maximal lifespan (Figure 3A and Table S3 in File S2). This short-lived phenotype due to loss of R148.3 was not associated with variations in the expression of the early development genes *wrt-2*, *abu-11*, or *lin-42* (Figure S3 in File S1). Importantly, a robust reduction in lifespan also occurred when RNAi was initiated at stage L4/young adult (Figure 3B and Table S4 in File S2), indicating postdevelopmental effects of R148.3 downregulation. In addition, R148.3 RNAi shortened the lifespan of worms deleted for the master regulator of lipid metabolism *sbp-1/SREBP* (Figure 3C and Table S5 in File S2), suggesting that the effects of R148.3 on survival and lipids are uncoupled.

**Figure 4:**
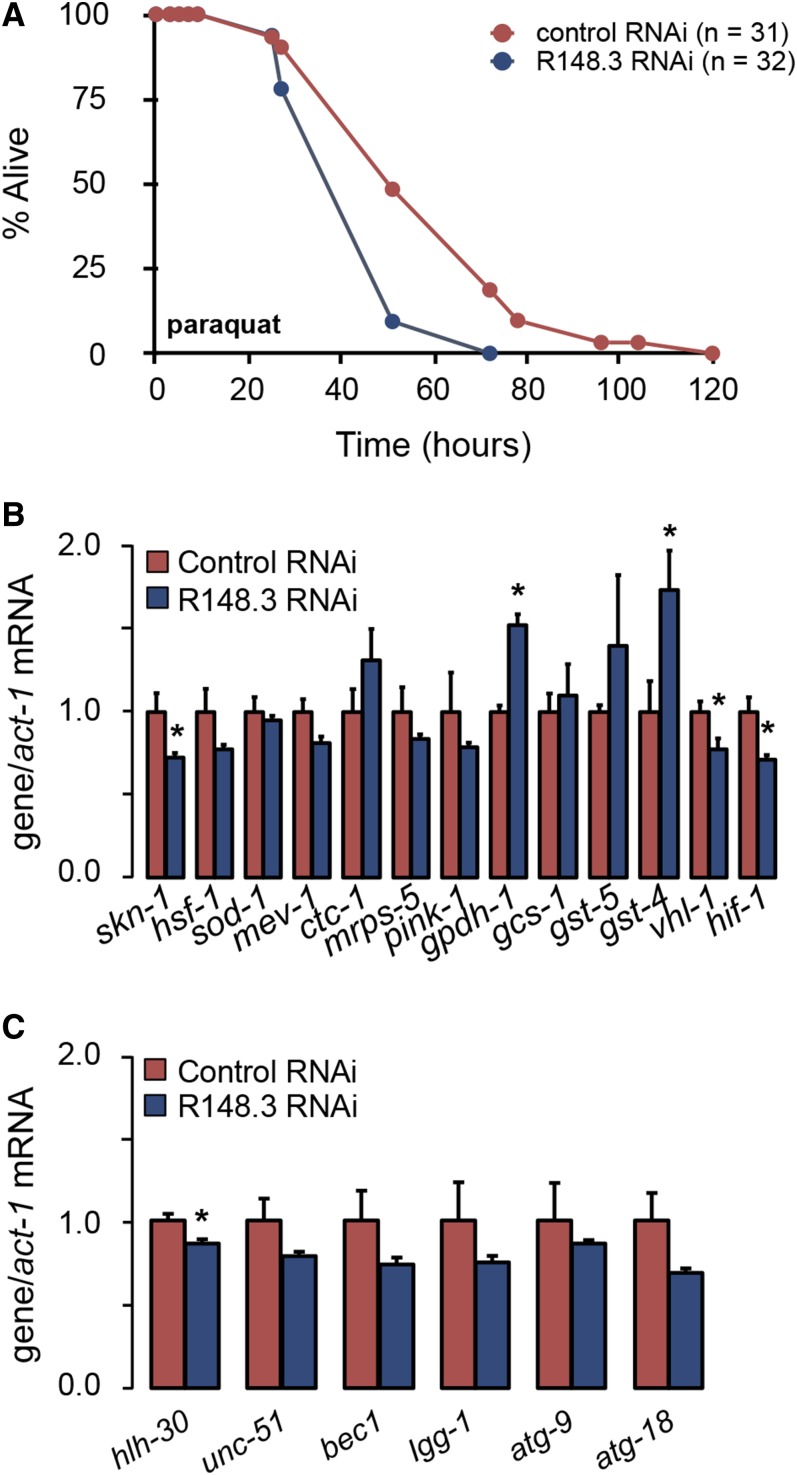
Loss of R148.3 increases susceptibility to oxidative stress. (A) Representative survival curve of adult N2 worms fed either empty vector or R148.3 RNAi, and treated with 100 mM paraquat. See Table S6 in File S2 for additional data on replicate experiments. (B) mRNA levels of genes involved in oxidative stress resistance in adult worms fed either empty vector or R148.3 RNAi. (C) mRNA levels of genes involved in autophagy in *control*(*RNAi*) and *R148.3*(*RNAi*) worms described in (B). For all panels, * *P* < 0.05.

Impaired stress resistance is a possible cause of shortened lifespan. Compared to *control*(*RNAi*) worms, *R148.3*(*RNAi*) worms showed higher sensitivity to oxidative stress, *i.e.*, enhanced mortality (reduction in mean and maximum lifespan by 13 and 25%, respectively) upon exposure to paraquat (Figure 4A and Table S6 in File S2). Depletion of R148.3 reduced the expression of oxidative stress resistance genes *skn-1/Nrf*, *vhl-1*, and *hif-1*, but increased the expression of *gpdh-1* and *gst-4* (Figure 4B). No major change was observed in the mRNA levels of genes involved in autophagy, except for a slight but significant downregulation of *hlh-30/TFEB* (Figure 4C). Loss of R148.3 did not modify the susceptibility of survival to high heat (35°) (data not shown), suggesting that it is required for some but not all types of stress.

**Figure 5:**
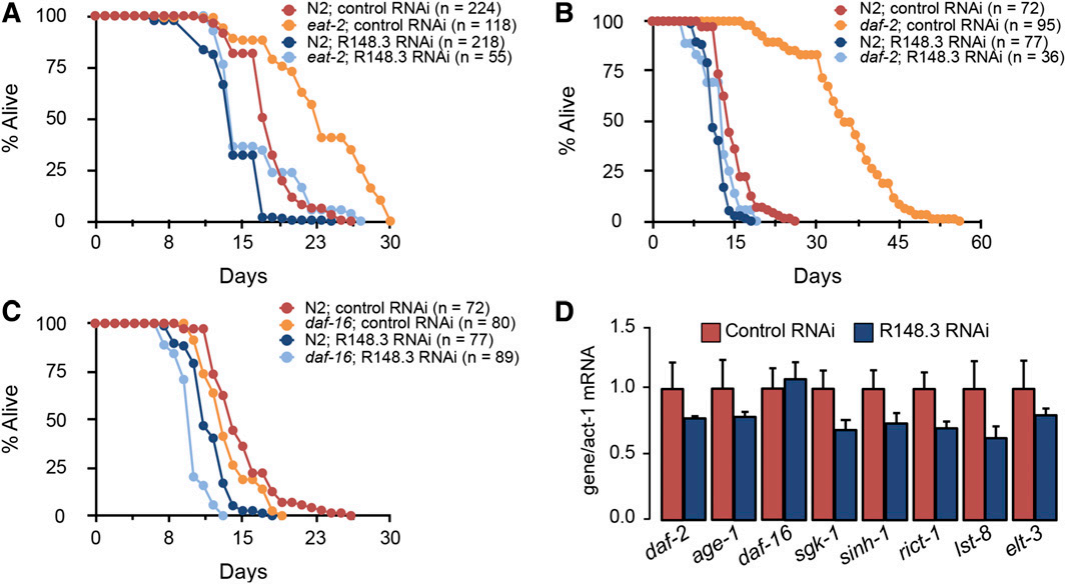
Loss of R148.3 blunts the long-lived phenotype of *eat-2* and *daf-2* mutants. (A) Representative population survival curve of N2 worms and *eat-2* mutants fed either empty vector or R148.3 RNAi (*P* < 0.0001). (B) Representative population survival curve of N2 worms and *daf-2* mutants and fed either empty vector or R148.3 RNAi (*P* < 0.0001). (C) Survival curve of N2 or *daf-16* mutants fed either empty vector or R148.3 RNAi (*P* < 0.0001). (D) mRNA expression levels of genes involved in the IISP in adult worms fed either empty vector or R148.3 RNAi. See Tables S7–S9 in File S2 for additional data on replicate survival experiments.

Finally, we investigated the life-shortening impact of R148.3 in long-lived mutants. As expected, *eat-2* and *daf-2/InsR* mutants showed a long-lived phenotype (Figure 5, A and B). Notably, R148.3 RNAi shortened the long lifespan of an *eat-2* mutant (Figure 5A and Table S7 in File S2), and it completely abrogated the effect of *daf-2* ablation on longevity (Figure 5B and Table S8 in File S2). In contrast, R148.3 was not required for the characteristic dauer trait of *daf-2* mutants (Figure S4 in File S1). R148.3 was also necessary for the life-extending effect of *age-1/PI3K* depletion (data not shown). Surprisingly, the decrease in lifespan associated with R148.3 RNAi remained effective in *daf-16*/FOXO mutants (Figure 5C and Table S9 in File S2). Depletion of R148.3 did not significantly alter the expression of genes involved in the insulin/insulin-like signaling pathway (IISP) (Figure 5D). Moreover, R148.3 RNAi did not appear to affect the levels and localization of a DAF-16::GFP fusion protein reporter (Figure S3B in File S1). These observations suggest that R148.3 influences longevity in part by interacting with the IISP pathway but independently of the key downstream effector *daf-16*.

## Discussion

In several species, the interactions between excess energy storage and longevity are complex, often resulting in U-shaped relationships. The present study demonstrates that the gene R148.3, likely coding for a secreted protein, has a profound impact on lipid accretion as well as lifespan. RNAi depletion of R148.3 at an early but not late larvae stage increased whole-body triglyceride content. However, downregulation of R148.3 drastically shortened lifespan, notwithstanding its time of induction, and impaired oxidative stress responses. Importantly, our findings suggest that R148.3 is required for the long-lived phenotypes of *daf-2* and *eat-2* mutants, implicating it as a novel player in several longevity pathways.

### Control of longevity by R148.3

The first important finding of our work is that R148.3 RNAi robustly shortened the lifespan of *C. elegans*, notwithstanding whether RNAi feeding was initiated at L1 or L4/young-adult stages. The postdevelopmental effects strongly suggest that loss of R148.3 does not impact viability by affecting crucial developmental pathways that alter health later in life. However, this possibility needs to be experimentally validated through analysis of longevity following R148.3 overexpression.

After 1 wk post-RNAi induction, worms fed R148.3 RNAi started to move less, which could be due to a decrease in body muscle function, as we observed that R148.3 is expressed in this tissue. R148.3 also appears to be expressed in neurons, as indicated by the R148.3*p*::GFP reporter. However, this might not contribute significantly to the life-span phenotype, as neurons are refractory to RNAi. Thus, direct effects of R148.3 on the neuronal regulation of body movements or longevity are unlikely. In contrast, the observation that food pumping was increased in *R148.3*(*RNAi*) worms, a process controlled centrally, suggests that a peripheral signal controlled by R148.3 may influence food intake, which in turn could modulate longevity.

The IISP is a prominent and conserved endocrine input regulating longevity. In *C. elegans*, mutations in the *daf-2* and *age-1* genes significantly increase longevity (Kenyon 2010). This is mediated through changes in the activity of few downstream effectors; including *daf-16*, *skn-1*, *hsf-1*, and *wwp-1*; which modulate oxidative and hypertonic stress, energy metabolism, detoxification, and pathogen resistance (Mallo *et al.* 2002; Murphy *et al.* 2003; Dorman *et al.* 1995; Kenyon *et al.* 1993; Chen *et al.* 2010; Lamitina and Strange 2005). Strikingly, depletion of R148.3 in *daf-2* mutants completely suppressed the long lifespan of these worms, suggesting that R148.3 is required as a downstream effector of IISP. However, because R148.3 is also required for the life-extending effect of the *eat-2* mutation, it is likely that R148.3 does not exclusively interact with the IISP, and thus should be studied in other long-lived mutants. Interestingly, loss of R148.3 did not impact the dauer-formation phenotype of *daf-2* mutants. Thus, interactions of R148.3 with the control of longevity does not seem to be specific for insulin signaling. The nature of these specific interactions remains to be investigated.

Although chronic activation of *daf-16* is responsible for the long life of *daf-2* mutants (Dorman *et al.* 1995), we found that *daf-16* is not required for the life-shortening effect of R148.3 depletion; suggesting that R148.3 might act in a *daf-16*-independent manner. This is consistent with the finding that R148.3 depletion does not alter levels and localization of a DAF-16::GFP fusion protein. This suggests that R148.3 exploits effectors downstream of *daf-2/age-1* but not *daf-16*.

Aging is associated with an accumulation of oxidative damage inside cells that may contribute to the development of age-related diseases. Reactive oxygen species (ROS) are metabolic byproducts, and organisms have developed adaptive responses to cope with ROS. For instance, long-lived *age-1* mutants are more resistant to oxidative damage than wild-type controls (Yanase *et al.* 2002). In agreement with this, we observed that *R148.3*(*RNAi*) worms displayed reduced expression of *skn-1* and *hif-1*, which likely contributed to their increased susceptibility to paraquat-induced oxidative stress. Whether this excessive oxidative stress or other processes involved in mitochondrial function [*e.g.*, mitophagy (Palikaras *et al.* 2015)] are responsible for the shortened lifespan upon loss of R148.3 is still undetermined. However, genetic or functional R148.3–*skn-1* interactions might contribute to this phenotype, as *skn-1*, like R148.3, is required for life-span control but dispensable for dauer formation (Tullet *et al.* 2008). This hypothesis requires to be validated by thorough experimentation.

### Effects of R148.3 on lipid metabolism

The second main finding of this work is that RNAi-induced depletion of R148.3 resulted in increased whole-body triglyceride content when RNAi feeding was initiated at the L1 stage. This phenotype, assessed by Oil Red O and Nile Red staining as well as by direct measurement of body triglyceride levels, manifested at the L4 stage and persisted during adulthood. Despite their importance in maintaining normal lipid levels (Lapierre *et al.* 2013), autophagy genes were not robustly altered by R148.3 RNAi, suggesting little contribution of this pathway to lipid accumulation in *R148.3*(*RNAi*) worms. As opposed to Oil Red O, which stains triglycerides in the hypodermis, gonads, and the intestine; Nile Red primarily stains lysosomal-related organelles (O’Rourke *et al.* 2009). The contribution of lipid signals originating from the lysosomes to the phenotype of R148.3-depleted worms might be of importance, especially since *lipl-4* expression was reduced by >50% in these animals in conjunction with increased Nile Red staining. The life-extending lysosomal lipase *lipl-4* was shown to stimulate oleoylethanolamide production, which promoted *nhr-49/nhr-80* transcriptional activity for expression of *acs-2* (Folick *et al.* 2015), an enzyme required for mitochondrial β-oxidation (Van Gilst *et al.* 2005). Because loss of R148.3 was associated with a 75% reduction in *acs-2* expression, it is plausible that diminished β-oxidation of fatty acids may have caused, at least in part, the increase in lipid levels observed in *R148.3*(*RNAi*) worms. This could be due to impaired NHR-80 levels and/or activity, since we observed no change in *nhr-49* levels or localization of a NHR-49::GFP reporter. More work is required to investigate whether the reduction in *lipl-4* is required for the effects of R148.3 on fat metabolism and longevity.

In contrast, when R148.3 RNAi was initiated at the L4/young-adult stage, we observed no effect on triglyceride levels. This suggests that R148.3 mainly impacts necessary early events involved in the control of lipid metabolism, and that loss of R148.3 during that specific period impairs this control. The molecular pathways involved remain to be determined. Importantly, despite unaltered fat accumulation upon R148.3 RNAi from stage L4 onwards, R148.3 depletion still reduced longevity, strongly suggesting that R148.3 affects the two processes independently. The fact that the loss of *sbp-1*, a factor essential for lipid synthesis (Yang *et al.* 2006), did not modify the impact of R148.3 RNAi on lifespan, also supports the notion that the modulation of survival and fat mass by R148.3 is dissociated.

The pharyngeal pumping rate was significantly higher upon R148.3 depletion, which is noteworthy because of the strong expression of R148.3 in the pharynx. As oxygen consumption, a direct measure of energy expenditure, was unchanged upon R148.3 depletion; the increased food intake likely contributes to enhanced triglyceride levels in *R148.3*(*RNAi*) worms. However, R148.3 was required for the long-lived phenotype of *eat-2* mutants. Since we did not measure pharyngeal pumping in *eat-2* worms, whether the effects of R148.3 on longevity and fat accumulation are uncoupled from energy intake needs to be further clarified.

Interestingly, detailed metabolomic analysis revealed that, whereas the total levels of acylcarnitines, glycerophospholipids, and sphingolipids were not altered, those of glycine and hexoses were robustly upregulated upon loss of R148.3. This could be caused by an overly saturated process of fat conversion from amino acid and carbohydrate precursors in conjunction with dysfunctional lipid oxidation. This could have triggered ectopic fat deposition in the hypodermis and the intestine, inducing lipotoxicity and exacerbating oxidative stress (Ackerman and Gems 2012). Interestingly, a recent study showed that modulation of ectopic fat deposition in heterologous tissues requires *hlh-30* (Palikaras *et al.* 2017). On the basis of this discovery, it is thus possible that the reduction in *hlh-30* in *R148.3*(*RNAi*) worms, although modest in nature, could have, in part, contributed to their fat phenotype. This hypothesis is plausible because HLH-30 impacts lysosomal lipolysis (O’Rourke and Ruvkun 2013), a process that was likely affected due to reduced *lipl-4* levels. In addition, previous work identified *skn-1* as a protective factor against high glucose-induced fat accumulation in *C. elegans* (Pang *et al.* 2014). Thus, since *R148.3*(*RNAi*) worms displayed reduced *skn-1* expression, it would be interesting to unravel the potential role of *skn-1* and its nuclear coactivator *mdt-15* (Goh *et al.* 2014) in the alterations in lipid oxidation and longevity caused by loss of R148.3.

### Nature of R148.3 as a secreted protein

The molecular action of R148.3 remains unknown. Sequence homology analyses suggest that R148.3 is an ortholog of the human FK506 binding protein 15 (FKBP15) gene, recently implicated in ulcerative colitis (Pan *et al.* 2015). Yeast two-hybrid screens to identify protein-protein interactions suggest that the PDZ domains of UNC-10 and PAT-12 may bind the proline-rich sequence of R148.3 (Lenfant *et al.* 2010), usually a characteristic of SH3-rich proteins, but these findings need validation. Importantly, our results indicate that R148.3 encodes a secreted protein. This possibility is consistent with a whole-genome survey of proteins with a signal peptide in *C. elegans* (Suh and Hutter 2012). Indeed, a mCherry protein fused with a putative SS in the N-terminus of R148.3 accumulated in coelomyocytes, which are scavenger-like cells continuously performing endocytosis of peptides circulating in the worm’s body cavity (Fares and Greenwald 2001). Moreover, large-scale proteomics in *C. elegans* revealed that R148.3 is *N*-glycosylated (Kaji *et al.* 2007). Since R148.3 does not have transmembrane domains, this also supports the notion that it is most likely secreted, although the probability that R148.3 could also act as an intracellular protein cannot be entirely ruled out. We note, however, that it is unclear what tissue(s) might be secreting the endogenous R148.3 protein. Our data indicate that R148.3 is widely and continuously expressed, which agrees with previous quantitative expression data from mENCODE libraries (http://wormbase.org). However, we note that the putative promoter used (1.5-kb upstream of GFP) may not represent the full expression pattern of the protein. In our experiments, we drove expression of SS_R148.3_::mCherry from the body wall muscle-specific *myo-3* promoter, but endogenous R148.3 promoter-driven expression must also be explored, since it is expressed in multiple tissues representing possible secretion sites. In sum, the precise tissues of expression, cellular targets of action, and interacting partners of R148.3 remain unidentified.

### Conclusions

Altogether, our findings demonstrate that R148.3 is a novel modulator of *C. elegans* lifespan and lipid metabolism by controlling lipid storage, food intake, and oxidative stress. Our data further suggest that R148.3 has broad effects, including interactions with the IISP, possibly as a secreted protein. More research is needed to understand how R148.3 independently controls fat deposition and longevity.

## Supplementary Material

Supplemental material is available online at www.g3journal.org/lookup/suppl/doi:10.1534/g3.117.041681/-/DC1.
