# Supplementary material for: The R148.3 Gene Modulates Caenorhabditis elegans Lifespan and Fat Metabolism
Source: G3 (Bethesda). 2017 Jun 15;7(8):2739–47. doi: 10.1534/g3.117.041681 (PMC5555478; doi:10.1534/g3.117.041681)
Supplement: Supplementary file 1 [file 2739FileS1.pdf]

Suppl Figure 1

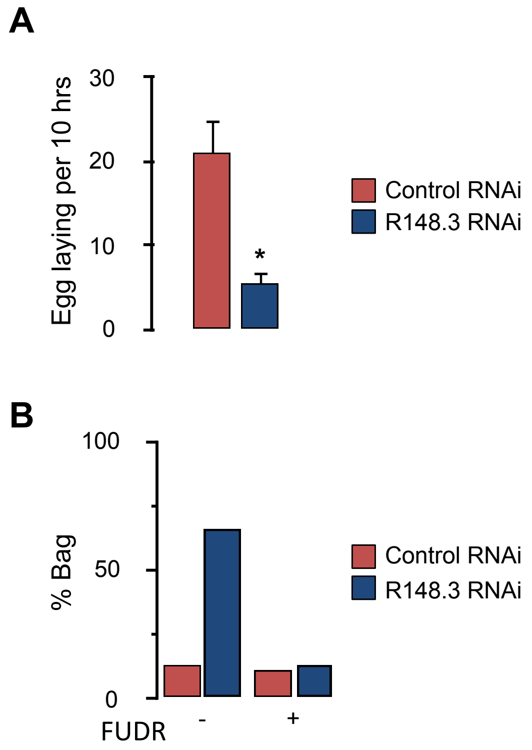

Figure S1 **Reduced R148.3 function induces a *Bag* phenotype.**

(A) Egg laying rate displayed by *control(RNAi)* or *R148.3(RNAi)* worms ($p < 0.001$). (B) Number of worms treated with either empty vector or R148.3 RNAi demonstrating a *Bag* phenotype with or without 50μM fluorodeoxyuridine (FUdR).

Suppl Figure 2

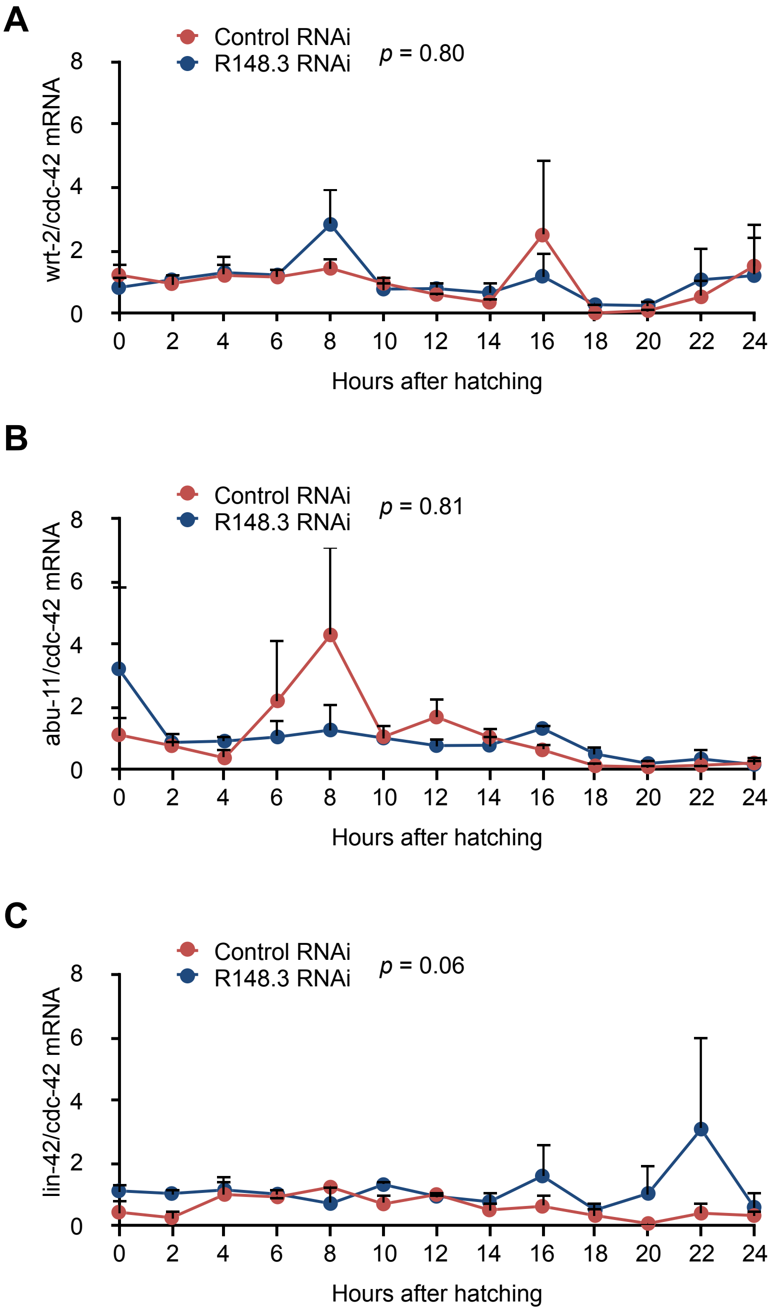

Figure S2 **Loss of R148.3 does not influence expression of early development genes.**

(A) *wrt-2*, (B) *abu-11*, and (C) *lin-42* mRNA expression levels in F2+ progeny of parent worms fed either empty vector or R148.3 RNAi, as determined by qPCR.

Suppl Figure 3

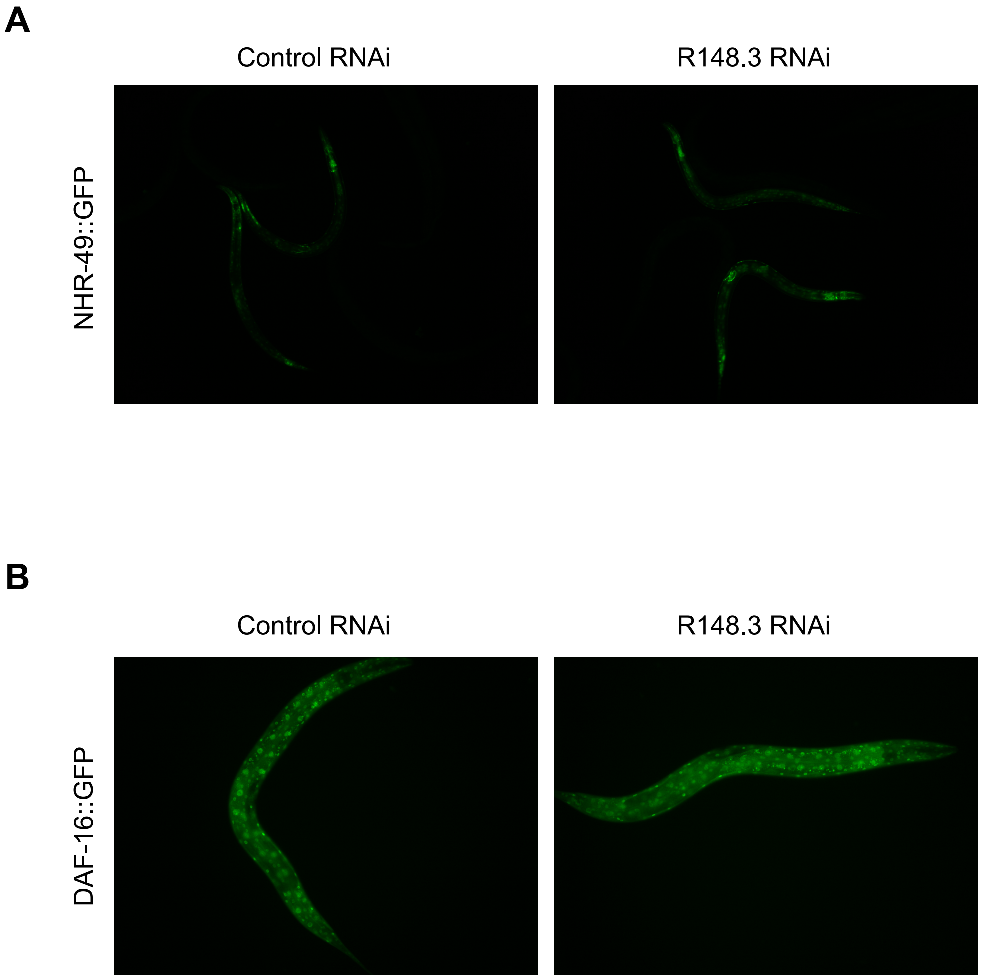

Figure S3 **Effects of R148.3 RNAi on NHR-49::GFP and DAF-16::GFP protein levels and localization.**

Representative immunofluorescence on NHR-49::GFP (A) and DAF-16::GFP (B) in L4 transgenic worms treated with either control RNAi or R148.3 RNAi.

Suppl Figure 4

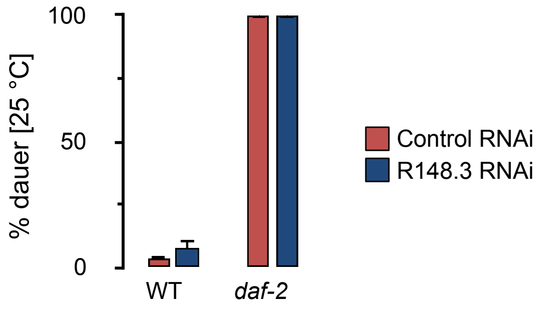

Figure S4 Loss of R148.3 does not impact the dauer trait of *daf-2* mutants.

Percentage of synchronized wild-type (WT) and *daf-2* worms treated with either empty vector or R148.3 RNAi showing a dauer phenotype when incubated at 25 ˚C at L1 stage. Data represent mean ± S.E.M. of three independent experiments. n = 673 (WT, control RNAi); 680 (WT, R148.3 RNAi); 661 (*daf-2*, control RNAi); 753 (*daf-2*, R148.3 RNAi).
