# Supplementary material for: The R148.3 Gene Modulates Caenorhabditis elegans Lifespan and Fat Metabolism
Source: G3 (Bethesda). 2017 Jun 15;7(8):2739–47. doi: 10.1534/g3.117.041681 (PMC5555478; doi:10.1534/g3.117.041681)
Supplement: Supplementary file 2 [file 2739FileS2.docx]

**SUPPLEMENTARY TABLES**

**Table S1**. Primer sequences used in this study.

| **Gene** | **Forward** | **Reverse** |
| --- | --- | --- |
| R148.3 | GACAACTCCAGCTCCTCTGG | CTCGGGCTCTTCAACTTTTG |
| *act-1* | GTCGGTATGGGACAGAAGGA | GGCTTCAGTGAGGAGGACTG |
| *nhr-49* | CAGAAGCCAAAGGACTCTCG | CGAGAATGAACTCGGAGAGC |
| *mdt-15* | ACCACCTGGTAGTGGTGCTC | ACCGTTCATTGGTCTTCCAG |
| *pod-2* | TCAGAAGTGCCGAGAACACC | ACGGAGACTTGAGCACACTG |
| *fasn-1* | GATCCATTTGCAACTGATTCC | GCTTGGTAAGGATGGTGGC |
| *lipl-1* | ATCGGTTTGCGCTGGACTTA | AGTTCCTGCGGGTGTATGTG |
| *lipl-3* | GCACGGACTTTTGTGTGCAT | GCCAAGCCACACATCGAATC |
| *lipl-5* | ACAGTTGCCACGGATGATG | TGTTGCATGAAGACGACAGG |
| *lipl-4* | GGAGTGATGATGATTGGCTTTC | CCCAAATGAAATGTAAGTGGTTG |
| *maoc-1* | GGGCTGGAAATGATTCTGAC | CCAATGTTTGTCCTGGAAGC |
| *dhs-28* | CGTTTCTTTCCTCAAGATGACC | CAGCGTAGTTGGCTTGTCC |
| *daf-22* | TGCTGTGGTCAACGTGCTC | CCCGGAGCCATGCGCTC |
| *prx-10* | TGAGCAGGGTCAGCAAAGAG | ACGTATTCCTCGCCAAGAGTT |
| *acs-2* | ACAGCTACATCCACGGAACC | TGACGGACGTCATGGTAGAA |
| *skn-1* | CTTCAGGACGTCAACAGCAG | GATTCCGAAGAGAGGCGAGA |
| *hsf-1* | ACGGAGCCAATGAGCAAAAG | CCCGAATAGTCTTGTTGCGG |
| *sod-1* | ATTTCTGCCGGTCCACACTT | ACCATAGATCGGCCAACGAC |
| *mev-1* | CTTCTCGTCGGAGGAATCGG | TGGCGAGAGCAAGAATAGCC |
| *ctc-1* | GGTGGGTTGACAGGTGTTGT | ACCCGTGTAGTCCTGCAAAA |
| *mrps-5* | GCCCACGTGGATTCGGTCTTA | GTTCGGCCAGTTGTTGATGAGTCT |
| *pink-1* | TGGCTCCATATCCGAATGCT | GTTTTCGGTTCGGAGGCAAT |
| *gpdh-1* | AGGATATCGAGAGGGCGTTG | AAGCTCCACGGCCTCATAAT |
| *gcs-1* | TGACGAAAGGTAGTCCGTTG | TTGAGCAATTCCGTGCTTC |
| *gst-5* | TCTCTTGCCGATCAATACAAGG | CCATGACAGCGTAGAAATATGG |
| *gst-4* | GATGCTCGTGCTCTTGCTG | CCGAATTGTTCTCCATCGAC |
| *vhl-1* | AGAAGTATTCTGGCCGGAGC | AGGAGGAGGAATTGAACGGC |
| *hif-1* | ACTTGCAGAGGGCATCTCAC | GCTTCCGATGACTGGGTTGA |
| *hlh-30* | ATCGCATCTTCCACCGATCC | GCGGTGTTGTTTGTCACCTC |
| *unc-51* | CATGATGCTTCACACCTTGG | ACAAATCCCTGTCGTTCCAG |
| *bec-1* | TGCAGTTGCTCGAGTTTTTG | TGACACCATTGTCAACCAGTG |
| *Igg-1* | AATGGGACAACTCTACCAGGAC | TCGGCGGATAATACATGACAC |
| *atg-9* | ACGGTTGAGCATTCATCAGG | TGGGAAGATAGGCGATTGG |
| *atg-18* | AGCCGCAAGGAGTAATCAAG | TCCAACTCTCCACCTTCTGG |
| *daf-2* | TGGCGTGAGAATGAAGTGAG | CGTCTCTTTCGGATCTTTCG |
| *age-1* | ATGGAGTTGCCTGAGCTGTC | ATCCATTGAAGGCTTCTTCG |
| *daf-16* | CTTTCCGTCCCCGAACTCAA | CGATCCACGGACACTGTTCA |
| *sgk-1* | GGGCCATCGCAATGATTTCC | GCGATTGGTGTCGACGAATG |
| *sinh-1* | CACCACAGACCCTTCCAGAC | GAGCTCGTGTTCGATTTCGC |
| *rict-1* | TTTGGCTGTACTGGCGTCAT | CCGAACCTCATCCTCATCCG |
| *Ist-8* | TGGCGTGGAGAAACAAAATGA | CGAACTTTCTGCCATGCACC |
| *elt-3* | CAACAGCTCACCAACGCATC | TTCTCCATTTCGTCGCCACA |
| *wrt-2* | AGGATCGCGGAAACGCTATT | TCTCCACAGCTTGCTCTTCG |
| *abu-11* | AAACCCAGTGCAACCAGGAA | GGACTGACGAGCAACTGGAA |
| *lin-42* | CAGATCAGGAAAGTTGCCAGC | GACATCGTCGGATAGGCGTT |
| *cdc-42* | CTGCTGGACAGGAAGATTACG | CTCGGACATTCTCGAATGAAG |

**Table S2.** Whole-body metabolomic analysis of worms fed either empty vector (control RNAi) or R148.3 RNAi. Data are mean ± S.E.M. of three independent experiments each performed on approximately 100 L4 worms. Results are expressed as µM/µg DNA. Only statistically significant *p* values are shown. Abbreviations: PC: Phosphatidylcholine; SM: Sphingolipid; SDMA: Symmetric dimethylarginine; ADMA: Asymmetric DMA; alpha-AAA: alpha-Aminoadipic acid; PEA: Phenylethylamine

| Acylcarnitines |  | Control RNAi | R148.3 RNAi | *p* |
| --- | --- | --- | --- | --- |
|  | C0 | 1.91 ± 0.44 | 1.57 ± 0.39 |  |
|  | C10 | 0.01 ± 0.01 | 0.01 ± 0.00 |  |
|  | C10:1 | 0.03 ± 0.01 | 0.03 ± 0.01 |  |
|  | C10:2 | 0.00 ± 0.00 | 0.01 ± 0.00 |  |
|  | C12 | 0.01 ± 0.00 | 0.01 ± 0.00 |  |
|  | C12-DC | 0.01 ± 0.00 | 0.01 ± 0.00 |  |
|  | C12:1 | 0.02 ± 0.00 | 0.02 ± 0.01 |  |
|  | C14 | 0.01 ± 0.00 | 0.01 ± 0.01 |  |
|  | C14:1 | 0.00 ± 0.00 | 0.00 ± 0.00 |  |
|  | C14:1-OH | 0.00 ± 0.00 | 0.00 ± 0.00 |  |
|  | C14:2-OH | 0.00 ± 0.00 | 0.00 ± 0.00 |  |
|  | C16 | 0.01 ± 0.00 | 0.01 ± 0.00 |  |
|  | C16-OH | 0.00 ± 0.00 | 0.00 ± 0.00 |  |
|  | C16:1 | 0.01 ± 0.00 | 0.02 ± 0.01 |  |
|  | C16:1-OH | 0.01 ± 0.00 | 0.00 ± 0.00 |  |
|  | C16:2 | 0.00 ± 0.00 | 0.00 ± 0.00 |  |
|  | C16:2-OH | 0.01 ± 0.00 | 0.01 ± 0.00 |  |
|  | C18 | 0.01 ± 0.00 | 0.01 ± 0.00 |  |
|  | C18:1 | 0.01 ± 0.00 | 0.01 ± 0.00 |  |
|  | C18:1-OH | 0.00 ± 0.00 | 0.00 ± 0.00 |  |
|  | C18:2 | 0.00 ± 0.00 | 0.01 ± 0.00 |  |
|  | C2 | 0.13 ± 0.03 | 0.15 ± 0.03 |  |
|  | C3 | 0.08 ± 0.00 | 0.10 ± 0.03 |  |
|  | C3-DC (C4-OH) | 0.01 ± 0.00 | 0.01 ± 0.00 |  |
|  | C3-OH | 0.01 ± 0.00 | 0.01 ± 0.00 |  |
|  | C3:1 | 0.00 ± 0.00 | 0.00 ± 0.00 |  |
|  | C4 | 0.02 ± 0.00 | 0.03 ± 0.00 |  |
|  | C4:1 | 0.00 ± 0.00 | 0.00 ± 0.00 |  |
|  | C5 | 0.05 ± 0.01 | 0.09 ± 0.02 |  |
|  | C5-DC (C6-OH) | 0.00 ± 0.00 | 0.00 ± 0.00 |  |
|  | C5-M-DC | 0.01 ± 0.00 | 0.01 ± 0.00 |  |
|  | C5-OH | 0.02 ± 0.00 | 0.02 ± 0.00 |  |
|  | C5:1 | 0.01 ± 0.00 | 0.01 ± 0.00 |  |
|  | C5:1-DC | 0.01 ± 0.00 | 0.00 ± 0.00 |  |
|  | C6 | 0.01 ± 0.00 | 0.01 ± 0.00 |  |
|  | C6:1 | 0.00 ± 0.00 | 0.00 ± 0.00 |  |
|  | C7-DC | 0.01 ± 0.00 | 0.01 ± 0.00 |  |
|  | C8 | 0.01 ± 0.01 | 0.01 ± 0.00 |  |
|  | C9 | 0.00 ± 0.00 | 0.01 ± 0.00 |  |
| TOTAL |  | 2.49 ± 0.53 | 2.26 ± 0.45 |  |

| Glycerophospholipids |  | Control RNAi | R148.3 RNAi | *p* |
| --- | --- | --- | --- | --- |
|  | lysoPC a C14:0 | 0.72 ± 0.19 | 0.71 ± 0.28 |  |
|  | lysoPC a C16:0 | 0.31 ± 0.09 | 0.31 ± 0.05 |  |
|  | lysoPC a C16:1 | 0.07 ± 0.01 | 0.08 ± 0.02 |  |
|  | lysoPC a C17:0 | 0.14 ± 0.09 | 0.10 ± 0.05 |  |
|  | lysoPC a C18:0 | 0.17 ± 0.07 | 0.16 ± 0.04 |  |
|  | lysoPC a C18:1 | 0.40 ± 0.05 | 0.39 ± 0.01 |  |
|  | lysoPC a C18:2 | 0.50 ± 0.17 | 0.45 ± 0.12 |  |
|  | lysoPC a C20:3 | 0.24 ± 0.07 | 0.20 ± 0.03 |  |
|  | lysoPC a C20:4 | 0.37 ± 0.09 | 0.32 ± 0.04 |  |
|  | lysoPC a C24:0 | 0.18 ± 0.07 | 0.15 ± 0.03 |  |
|  | lysoPC a C26:0 | 0.09 ± 0.02 | 0.07 ± 0.01 |  |
|  | lysoPC a C26:1 | 0.21 ± 0.07 | 0.13 ± 0.02 |  |
|  | lysoPC a C28:0 | 0.09 ± 0.03 | 0.07 ± 0.01 |  |
|  | lysoPC a C28:1 | 0.22 ± 0.05 | 0.22 ± 0.05 |  |
|  | PC aa C24:0 | 0.04 ± 0.01 | 0.03 ± 0.01 |  |
|  | PC aa C26:0 | 0.17 ± 0.03 | 0.18 ± 0.06 |  |
|  | PC aa C28:1 | 0.07 ± 0.00 | 0.07 ± 0.01 |  |
|  | PC aa C30:0 | 0.14 ± 0.02 | 0.11 ± 0.01 |  |
|  | PC aa C30:2 | 0.07 ± 0.01 | 0.07 ± 0.00 |  |
|  | PC aa C32:0 | 0.20 ± 0.03 | 0.17 ± 0.03 |  |
|  | PC aa C32:1 | 1.03 ± 0.15 | 1.06 ± 0.18 |  |
|  | PC aa C32:2 | 0.64 ± 0.15 | 0.67 ± 0.13 |  |
|  | PC aa C32:3 | 0.26 ± 0.08 | 0.24 ± 0.06 |  |
|  | PC aa C34:1 | 1.78 ± 0.28 | 1.66 ± 0.07 |  |
|  | PC aa C34:2 | 3.52 ± 0.34 | 3.91 ± 0.10 |  |
|  | PC aa C34:3 | 3.29 ± 0.77 | 3.57 ± 1.05 |  |
|  | PC aa C34:4 | 0.70 ± 0.18 | 0.71 ± 0.15 |  |
|  | PC aa C36:0 | 0.91 ± 0.19 | 0.62 ± 0.09 |  |
|  | PC aa C36:1 | 0.53 ± 0.07 | 0.39 ± 0.05 |  |
|  | PC aa C36:2 | 2.95 ± 0.43 | 2.88 ± 0.10 |  |
|  | PC aa C36:3 | 3.24 ± 0.38 | 3.23 ± 0.20 |  |
|  | PC aa C36:4 | 3.01 ± 0.21 | 3.20 ± 0.31 |  |
|  | PC aa C36:5 | 3.98 ± 0.09 | 4.08 ± 0.37 |  |
|  | PC aa C36:6 | 1.26 ± 0.35 | 1.51 ± 0.38 |  |
|  | PC aa C38:0 | 0.33 ± 0.09 | 0.26 ± 0.05 |  |
|  | PC aa C38:1 | 0.08 ± 0.02 | 0.06 ± 0.01 |  |
|  | PC aa C38:3 | 0.60 ± 0.03 | 0.47 ± 0.04 | 0.0426 |
|  | PC aa C38:4 | 1.40 ± 0.21 | 1.45 ± 0.08 |  |
|  | PC aa C38:5 | 3.68 ± 0.13 | 3.40 ± 0.34 |  |
|  | PC aa C38:6 | 7.19 ± 0.66 | 7.19 ± 0.37 |  |
|  | PC aa C40:1 | 0.08 ± 0.02 | 0.07 ± 0.02 |  |
|  | PC aa C40:2 | 0.06 ± 0.02 | 0.04 ± 0.00 |  |
|  | PC aa C40:3 | 0.05 ± 0.01 | 0.04 ± 0.00 |  |
|  | PC aa C40:4 | 0.08 ± 0.01 | 0.07 ± 0.01 |  |
|  | PC aa C40:5 | 0.23 ± 0.03 | 0.18 ± 0.01 |  |
|  | PC aa C40:6 | 0.79 ± 0.08 | 0.72 ± 0.07 |  |
|  | PC aa C42:0 | 0.04 ± 0.01 | 0.03 ± 0.01 |  |
|  | PC aa C42:1 | 0.04 ± 0.01 | 0.04 ± 0.00 |  |
|  | PC aa C42:2 | 0.03 ± 0.01 | 0.02 ± 0.01 |  |
|  | PC aa C42:4 | 0.01 ± 0.00 | 0.01 ± 0.00 |  |
|  | PC aa C42:5 | 0.04 ± 0.01 | 0.03 ± 0.01 |  |
|  | PC aa C42:6 | 0.04 ± 0.01 | 0.04 ± 0.00 |  |
|  | PC ae C30:0 | 0.07 ± 0.00 | 0.05 ± 0.01 |  |
|  | PC ae C30:1 | 0.06 ± 0.01 | 0.05 ± 0.01 |  |
|  | PC ae C30:2 | 0.06 ± 0.01 | 0.06 ± 0.01 |  |
|  | PC ae C32:1 | 0.37 ± 0.07 | 0.30 ± 0.07 |  |
|  | PC ae C32:2 | 0.11 ± 0.01 | 0.12 ± 0.01 |  |
|  | PC ae C34:0 | 0.11 ± 0.02 | 0.07 ± 0.01 |  |
|  | PC ae C34:1 | 0.96 ± 0.08 | 0.97 ± 0.08 |  |
|  | PC ae C34:2 | 1.26 ± 0.08 | 1.49 ± 0.13 |  |
|  | PC ae C34:3 | 0.31 ± 0.00 | 0.37 ± 0.01 | 0.0117 |
|  | PC ae C36:0 | 0.32 ± 0.03 | 0.27 ± 0.02 |  |
|  | PC ae C36:1 | 1.05 ± 0.17 | 0.83 ± 0.04 |  |
|  | PC ae C36:2 | 2.80 ± 0.19 | 2.82 ± 0.22 |  |
|  | PC ae C36:3 | 2.79 ± 0.63 | 3.10 ± 0.86 |  |
|  | PC ae C36:4 | 1.10 ± 0.05 | 1.30 ± 0.17 |  |
|  | PC ae C36:5 | 1.66 ± 0.38 | 1.49 ± 0.27 |  |
|  | PC ae C38:0 | 4.85 ± 0.93 | 4.75 ± 1.23 |  |
|  | PC ae C38:1 | 1.22 ± 0.09 | 1.38 ± 0.03 |  |
|  | PC ae C38:2 | 1.00 ± 0.20 | 0.99 ± 0.18 |  |
|  | PC ae C38:3 | 1.38 ± 0.51 | 1.51 ± 0.65 |  |
|  | PC ae C38:4 | 1.30 ± 0.27 | 1.31 ± 0.15 |  |
|  | PC ae C38:5 | 2.80 ± 0.83 | 2.47 ± 0.48 |  |
|  | PC ae C38:6 | 3.42 ± 0.68 | 3.89 ± 0.54 |  |
|  | PC ae C40:1 | 2.01 ± 0.17 | 2.26 ± 0.16 |  |
|  | PC ae C40:2 | 3.21 ± 0.61 | 3.43 ± 0.51 |  |
|  | PC ae C40:3 | 5.71 ± 1.63 | 5.37 ± 1.51 |  |
|  | PC ae C40:4 | 0.45 ± 0.14 | 0.42 ± 0.10 |  |
|  | PC ae C40:5 | 1.01 ± 0.39 | 0.77 ± 0.14 |  |
|  | PC ae C40:6 | 3.72 ± 1.88 | 3.03 ± 1.24 |  |
|  | PC ae C42:0 | 0.11 ± 0.04 | 0.10 ± 0.03 |  |
|  | PC ae C42:1 | 0.04 ± 0.01 | 0.04 ± 0.01 |  |
|  | PC ae C42:2 | 0.04 ± 0.01 | 0.03 ± 0.00 |  |
|  | PC ae C42:3 | 0.06 ± 0.01 | 0.05 ± 0.00 |  |
|  | PC ae C42:4 | 0.02 ± 0.01 | 0.02 ± 0.00 |  |
|  | PC ae C42:5 | 0.18 ± 0.04 | 0.17 ± 0.06 |  |
|  | PC ae C44:3 | 0.01 ± 0.00 | 0.01 ± 0.00 |  |
|  | PC ae C44:4 | 0.01 ± 0.00 | 0.02 ± 0.00 |  |
|  | PC ae C44:5 | 0.04 ± 0.01 | 0.03 ± 0.01 |  |
|  | PC ae C44:6 | 0.03 ± 0.01 | 0.02 ± 0.00 |  |
| TOTAL |  | 89.81 ± 10.19 | 88.24 ± 8.91 |  |

| Sphingolipids |  | Control RNAi | R148.3 RNAi | *p* |
| --- | --- | --- | --- | --- |
|  | SM (OH) C14:1 | 0.09 ± 0.01 | 0.11 ± 0.01 |  |
|  | SM (OH) C16:1 | 0.04 ± 0.01 | 0.04 ± 0.00 |  |
|  | SM (OH) C22:1 | 0.06 ± 0.04 | 0.04 ± 0.02 |  |
|  | SM (OH) C22:2 | 0.07 ± 0.02 | 0.02 ± 0.01 |  |
|  | SM (OH) C24:1 | 0.07 ± 0.01 | 0.04 ± 0.02 |  |
|  | SM C16:0 | 0.17 ± 0.04 | 0.31 ± 0.11 |  |
|  | SM C16:1 | 0.05 ± 0.01 | 0.07 ± 0.02 |  |
|  | SM C18:0 | 0.04 ± 0.01 | 0.05 ± 0.02 |  |
|  | SM C18:1 | 0.01 ± 0.01 | 0.01 ± 0.01 |  |
|  | SM C20:2 | not detectable | not detectable |  |
|  | SM C22:3 | not detectable | not detectable |  |
|  | SM C24:0 | 0.17 ± 0.05 | 0.15 ± 0.04 |  |
|  | SM C24:1 | 0.03 ± 0.02 | 0.06 ± 0.03 |  |
|  | SM C26:0 | 0.00 ± 0.00 | 0.01 ± 0.00 |  |
|  | SM C26:1 | 0.00 ± 0.00 | 0.00 ± 0.00 |  |
| TOTAL |  | 0.79 ± 0.15 | 0.90 ± 0.23 |  |

| Hexoses |  | Control RNAi | R148.3 RNAi | *p* |
| --- | --- | --- | --- | --- |
|  | H1 | 52.17 ± 3.80 | 67.06 ± 3.37 | 0.0427 |

| Amino acids |  | Control RNAi | R148.3 RNAi | *p* |
| --- | --- | --- | --- | --- |
|  | Ala | 343.16 ± 50.93 | 419.96 ± 34.92 |  |
|  | Arg | 71.17 ± 10.72 | 67.13 ± 7.99 |  |
|  | Asn | 22.87 ± 3.44 | 25.31 ± 5.23 |  |
|  | Asp | 7.98 ± 3.09 | 12.32 ± 8.91 |  |
|  | Cit | 0.79 ± 0.38 | 1.35 ± 1.23 |  |
|  | Gln | 58.27 ± 2.39 | 69.48 ± 14.79 |  |
|  | Glu | 141.20 ± 26.42 | 156.95 ± 32.54 |  |
|  | Gly | 48.79 ± 3.57 | 77.56 ± 7.76 | 0.0281 |
|  | His | 15.24 ± 1.75 | 22.24 ± 5.91 |  |
|  | Ile | 19.44 ± 4.78 | 25.54 ± 6.17 |  |
|  | Leu | 38.32 ± 12.37 | 45.32 ± 14.38 |  |
|  | Lys | 36.68 ± 3.49 | 42.18 ± 2.73 |  |
|  | Met | 14.55 ± 5.09 | 15.72 ± 5.46 |  |
|  | Orn | 5.64 ± 1.79 | 9.84 ± 5.19 |  |
|  | Phe | 21.84 ± 2.61 | 26.49 ± 2.98 |  |
|  | Pro | 24.11 ± 3.99 | 32.51 ± 8.05 |  |
|  | Ser | 34.10 ± 1.58 | 59.46 ± 21.59 |  |
|  | Thr | 37.93 ± 11.87 | 48.05 ± 2.55 |  |
|  | Trp | 6.56 ± 0.98 | 8.22 ± 0.78 |  |
|  | Tyr | 17.34 ± 3.03 | 19.20 ± 2.94 |  |
|  | Val | 25.68 ± 5.12 | 31.03 ± 5.23 |  |

| Biogenic amines |  | Control RNAi | R148.3 RNAi | *p* |
| --- | --- | --- | --- | --- |
|  | Ac-Orn | 8.19 ± 0.90 | 7.77 ± 0.34 |  |
|  | ADMA | 0.22 ± 0.11 | 0.15 ± 0.10 |  |
|  | alpha-AAA | 17.88 ± 1.81 | 25.58 ± 3.96 |  |
|  | Carnosine | 0.36 ± 0.16 | 0.35 ± 0.13 |  |
|  | Creatinine | barely detectable | barely detectable |  |
|  | DOPA | barely detectable | barely detectable |  |
|  | Dopamine | barely detectable | barely detectable |  |
|  | Histamine | 0.07 ± 0.02 | 0.07 ± 0.03 |  |
|  | Kynurenine | 0.05 ± 0.02 | 0.06 ± 0.03 |  |
|  | Met-SO | 1.55 ± 0.16 | 2.08 ± 0.30 |  |
|  | Nitro-Tyr | barely detectable | barely detectable |  |
|  | OH-Pro | barely detectable | barely detectable |  |
|  | PEA | barely detectable | barely detectable |  |
|  | Putrescine | 16.32 ± 2.22 | 16.31 ± 2.60 |  |
|  | Sarcosine | barely detectable | barely detectable |  |
|  | SDMA | barely detectable | barely detectable |  |
|  | Serotonin | 0.01 ± 0.00 | 0.01 ± 0.00 |  |
|  | Spermidine | 5.69 ± 0.53 | 6.24 ± 0.60 |  |
|  | Spermine | barely detectable | barely detectable |  |
|  | Taurine | 0.36 ± 0.08 | 4.23 ± 3.96 |  |
|  | total DMA | 0.16 ± 0.09 | 0.17 ± 0.10 |  |

**For Tables S3 to S9:** n = total number of deaths that occurred during the assay, excluding censored worms (e.g. exploded through vulva, crawled off). *p* values calculated on mean lifespan by log-rank test.

**Table S3: Survival of N2 worms on control and R148.3 RNAi starting at L1**

| **Exp #** | **RNAi** | **n** | **Median lifespan (days)** | **Mean lifespan**  **(days) ± SEM** | ***p* value vs control RNAi** |
| --- | --- | --- | --- | --- | --- |
| 1 | control | 87 | 13 | 13.8 ± 0.3 | < 0.0001 |
|  | R148.3 | 68 | 12 | 10.5 ± 0.2 |  |
| 2  see Fig 3A | control | 108 | 15 | 15.3 ± 0.4 | < 0.0001 |
|  | R148.3 | 60 | 9 | 9.0 ± 0.3 |  |
| 3 | control | 45 | 19 | 19.2 ± 0.7 | < 0.0001 |
|  | R148.3 | 38 | 14 | 12.3 ± 0.5 |  |
| 4 | control | 67 | 18 | 16.9 ± 0.5 | < 0.0001 |
|  | R148.3 | 43 | 11 | 11.2 ± 0.6 |  |

**Table S4: Survival of N2 worms on control and R148.3 RNAi starting at L4/young adult (before egg formation)**

| **Exp #** | **RNAi** | **n** | **Median lifespan (days)** | **Mean lifespan**  **(days) ± SEM** | ***p* value vs control RNAi** |
| --- | --- | --- | --- | --- | --- |
| 1  see fig 3B | control | 256 | 19 | 20.0 ± 0.2 | < 0.0001 |
|  | R148.3 | 319 | 17 | 16.5 ± 0.1 |  |
| 2 | control | 336 | 22 | 21.8 ± 0.3 | < 0.0001 |
|  | R148.3 | 340 | 16 | 15.9 ± 0.1 |  |
| 3 | control | 332 | 19 | 19.9 ± 0.3 | < 0.0001 |
|  | R148.3 | 342 | 18 | 17.1 ± 0.2 |  |

**Table S5: Survival of N2 or *sbp-1* worms on control and R148.3 RNAi.**

| **Exp #** | **Strain** | **RNAi** | **n** | **Median lifespan (days)** | **Mean lifespan**  **(days) ± SEM** | ***p* value vs control RNAi** |
| --- | --- | --- | --- | --- | --- | --- |
| 1  see Fig 3C | N2 | control | 67 | 22 | 21.4 ± 0.5 | < 0.0001 |
|  | N2 | R148.3 | 71 | 15 | 15.0 ± 0.3 |  |
|  | *sbp-1* | control | 60 | 21 | 20.1 ± 0.4 | < 0.0001 |
|  | *sbp-1* | R148.3 | 47 | 11 | 10.6 ± 0.3 |  |
| 2 | N2 | control | 42 | 16 | 16.6 ± 0.4 | < 0.0001 |
|  | N2 | R148.3 | 31 | 13 | 11.9 ± 0.3 |  |
|  | *sbp-1* | control | 59 | 16 | 16.3 ± 0.5 | < 0.0001 |
|  | *sbp-1* | R148.3 | 53 | 10 | 10.1 ± 0.3 |  |

**Table S6: Survival of N2 worms or mutants on control and R148.3 RNAi upon 100 mM paraquat treatment.**

| **Exp #** | **RNAi** | **n** | **Median lifespan (hours)** | **Mean lifespan**  **(hours) ± SEM** | ***p* value vs control RNAi** |
| --- | --- | --- | --- | --- | --- |
| 1  see Fig 4A | control | 31 | 72 | 62.4 ± 3.7 | 0.0006 |
|  | R148.3 | 32 | 72 | 47.6 ± 2.3 |  |
| 2 | control | 32 | 54 | 56.1 ± 3.3 | 0.002 |
|  | R148.3 | 32 | 54 | 46.9 ± 1.5 |  |
| 3 | control | 32 | 72 | 69.4 ± 4.1 | 0.062 |
|  | R148.3 | 32 | 56 | 60.7 ± 3.5 |  |

**Table S7: Survival of N2 or *eat-2* worms on control and R148.3 RNAi.**

| **Exp #** | **Strain** | **RNAi** | **n** | **Median lifespan (days)** | **Mean lifespan**  **(days) ± SEM** | ***p* value vs control RNAi** |
| --- | --- | --- | --- | --- | --- | --- |
| 1 | N2 | control | 39 | 21 | 20.0 ± 0.8 | 0.0001 |
|  | N2 | R148.3 | 20 | 13 | 13.5 ± 1.0 |  |
|  | *eat-2* | control | 22 | 29 | 26.9 ± 1.2 | 0.001 |
|  | *eat-2* | R148.3 | 25 | 19 | 19.0 ± 0.9 |  |
| 2  see Fig 5A | N2 | control | 224 | 18 | 17.7 ± 0.2 | < 0.0001 |
|  | N2 | R148.3 | 218 | 14 | 14.2 ± 0.2 |  |
|  | *eat-2* | control | 118 | 22 | 22.0 ± 0.5 | < 0.0001 |
|  | *eat-2* | R148.3 | 55 | 15 | 16.2 ± 0.5 |  |

**Table S8: Survival of N2 or *daf-2* worms on control and R148.3 RNAi.**

| **Exp #** | **Strain** | **RNAi** | **n** | **Median lifespan (days)** | **Mean lifespan**  **(days) ± SEM** | ***p* value vs control RNAi** |
| --- | --- | --- | --- | --- | --- | --- |
| 1  see Fig 5B | N2 | control | 72 | 14 | 14.9 ± 0.4 | < 0.0001 |
|  | N2 | R148.3 | 77 | 12 | 11.7 ± 0.2 |  |
|  | *daf-2* | control | 95 | 35 | 35.4 ± 0.8 | < 0.0001 |
|  | *daf-2* | R148.3 | 36 | 13 | 12.4 ± 0.6 |  |
| 2 | N2 | control | 39 | 19 | 16.9 ± 0.9 | < 0.0001 |
|  | N2 | R148.3 | 47 | 13 | 11.8 ± 0.6 |  |
|  | *daf-2* | control | 29 | 35 | 34.0 ± 2.3 | < 0.0001 |
|  | *daf-2* | R148.3 | 33 | 20 | 21.1 ± 1.0 |  |
| 3 | N2 | control | 138 | 16 | 18.6 ± 0.6 | < 0.0001 |
|  | N2 | R148.3 | 137 | 9 | 12.9 ± 0.8 |  |
|  | *daf-2* | control | 130 | 32 | 31.2 ± 1.0 | < 0.0001 |
|  | *daf-2* | R148.3 | 136 | 9 | 10.1 ± 0.6 |  |

**Table S9: Survival of N2 or *daf-16* worms on control and R148.3 RNAi.**

| **Exp #** | **Strain** | **RNAi** | **n** | **Median lifespan (days)** | **Mean lifespan**  **(days) ± SEM** | ***p* value vs control RNAi** |
| --- | --- | --- | --- | --- | --- | --- |
| 1  see Fig 5C | N2 | control | 72 | 14 | 14.9 ± 0.4 | < 0.0001 |
|  | N2 | R148.3 | 77 | 12 | 11.7 ± 0.2 |  |
|  | *daf-16* | control | 80 | 13 | 13.5 ± 0.3 | < 0.0001 |
|  | *daf-16* | R148.3 | 89 | 10 | 9.9 ± 0.2 |  |
| 2 | N2 | control | 45 | 24 | 22.4 ± 0.7 | < 0.0001 |
|  | N2 | R148.3 | 36 | 13 | 12.4 ± 0.4 |  |
|  | *daf-16* | control | 21 | 16 | 16.1 ± 1.0 | 0.46 |
|  | *daf-16* | R148.3 | 16 | 13 | 13.8 ± 1.2 |  |
